# Search-based Automatic Repair for Fairness and Accuracy in Decision-making Software

**DOI:** 10.1007/s10664-023-10419-3

**Published:** 2024-01-03

**Authors:** Max Hort, Jie M. Zhang, Federica Sarro, Mark Harman

**Affiliations:** 1 Simula Research Laboratory, Oslo, Norway; 2 Kings College London, London, UK; 3 University College London, London, UK

**Keywords:** Software fairness, Bias mitigation, Classification, Multi-objective optimization

## Abstract

Decision-making software mainly based on Machine Learning (ML) may contain fairness issues (e.g., providing favourable treatment to certain people rather than others based on sensitive attributes such as gender or race). Various mitigation methods have been proposed to automatically repair fairness issues to achieve fairer ML software and help software engineers to create responsible software. However, existing bias mitigation methods trade accuracy for fairness (i.e., trade a reduction in accuracy for better fairness). In this paper, we present a novel search-based method for repairing ML-based decision making software to simultaneously increase both its fairness and accuracy. As far as we know, this is the first bias mitigation approach based on multi-objective search that aims to repair fairness issues without trading accuracy for binary classification methods. We apply our approach to two widely studied ML models in the software fairness literature (i.e., Logistic Regression and Decision Trees), and compare it with seven publicly available state-of-the-art bias mitigation methods by using three different fairness measurements. The results show that our approach successfully increases both accuracy and fairness for 61% of the cases studied, while the state-of-the-art always decrease accuracy when attempting to reduce bias. With our proposed approach, software engineers that previously were concerned with accuracy losses when considering fairness, are now enabled to improve the fairness of binary classification models without sacrificing accuracy.

## Introduction

Discrimination occurs when a decision about a person is made based on sensitive attributes such as race or gender rather than merit. This suppresses opportunities of deprived groups or individuals (e.g., in education, or finance) (Kamiran et al. 2012, 2018). While software systems do not explicitly incorporate discrimination, they are not spared from biased decisions and unfairness. For example, Machine Learning (ML) software, which nowadays is widely used in critical decision-making software such as software justice risk assessment (Angwin et al. 2016; Berk et al. 2018) and pedestrian detection for autonomous driving systems (Li et al. 2023) has shown to exhibit discriminatory behaviours (Pedreshi et al. 2008). Such discriminatory behaviours can be highly detrimental, affecting human rights (Mehrabi et al. 2019), profit and revenue (Mikians et al. 2012), and can also fall under regulatory control (Pedreshi et al. 2008; Chen et al. 2019; Romei and Ruggieri 2011). To combat this, software fairness aims to provide algorithms that operate in a non-discriminatory manner (Friedler et al. 2019) for humans.

Due to its importance as a non-functional property, software fairness has recently received a lot of attention, in the literature of software engineering (Zhang et al. 2020; Brun and Meliou 2018; Zhang and Harman 2021; Horkoff 2019; Chakraborty et al. 2020; Tizpaz-Niari et al. 2022; Hort et al. 2021; Chen et al. 2022b). Indeed, it is the duty of software engineers and researchers to create responsible software.

A simple approach for repairing fairness issues in ML software is the removal of sensitive attributes (i.e., attributes that constitute discriminative decisions, such as age, gender, or race) from the training data. However, this has shown to not be able to combat unfairness and discriminative classification, owing to correlation of other attributes with sensitive attributes (Kamiran and Calders 2009; Calders et al. 2009; Pedreshi et al. 2008). Therefore, more advanced methods have been proposed in the literature, which apply bias mitigation^1^ at different stages of the software development process. Bias mitigation has been applied before training software models (pre-processing) (Calmon et al. 2017; Feldman et al. 2015; Chakraborty et al. 2020; Kamiran and Calders 2012), during the training process (in-processing) (Zhang et al. 2018; Kearns et al. 2018; Celis et al. 2019; Berk et al. 2017; Zafar et al. 2017), and after a software model has been trained (post-processing) (Pleiss et al. 2017; Hardt et al. 2016; Calders and Verwer 2010; Kamiran et al. 2010, 2018). However, there are limitations for the applicability of these methods and it has been shown that they often reduce bias at the cost of accuracy (Kamiran et al. 2012, 2018), known as the *price of fairness* (Berk et al. 2017).

In this paper, we introduce the use of a **multi-objective search-based** procedure to **mutate** binary classification models in a post-processing stage, in order to automatically **repair software fairness and accuracy issues** and conduct a thorough empirical study to evaluate its feasibility and effectiveness. Here, binary classification models represent an important component of fairness research, with hundreds of publications addressing their fairness improvements (Hort et al. 2023a). We apply our method on two widely-studied binary classification models in ML software fairness research, namely Logistic Regression (Feldman et al. 2015; Chakraborty et al. 2020; Zafar et al. 2017; Kamiran et al. 2012; Kamishima et al. 2012; Kamiran et al. 2018) and Decision Trees (Kamiran et al. 2010, 2012, 2018; Žliobaite et al. 2011), which belong to two different families of classifiers. These two models are also widely adopted in practice on fairness-critical scenarios, mainly due to their advantages in explainability.^2^ We investigate the performance on four widely adopted datasets, and measure the fairness with three widely-adopted fairness metrics. Furthermore, we benchmark our method with all existing post-processing methods publicly available from the popular IBM AIF360 framework (Bellamy et al. 2018), as well as three pre-processing and one in-processing bias mitigation method.

The results show that our approach is able to improve both accuracy and fairness of Logistic Regression and Decision Tree classifiers in 61% of the cases. The three post-processing bias mitigation methods we studied conform to the fairness-accuracy trade-off and therefore decrease accuracy when attempting to mitigate bias. Among all post-processing repair methods, our approach achieves the highest accuracy in 100% of the cases, while also achieving the lowest bias in 33% of these. When compared to pre- and in-processing bias mitigation methods, our approaches show a better or comparable performance (i.e., they are not outperformed by the existing methods) in 87% of the evaluations. With our approach, engineers are able to develop fairer binary classification models without the need to sacrifice accuracy.

In summary, we make the following contributions:

- We propose a novel application of multi-objective search to debias classification models in a post-processing fashion.
- We carry out a thorough empirical study to evaluate the applicability and effectiveness of our search-based post-processing approach to two different classification models (Logistic Regression and Decision Trees) on four publicly available datasets, and benchmark it to seven state-of-the-art post-processing methods according to three fairness metrics.

Additionally, we make our scripts and experimental results publicly available to allow for replication and extension of our work (Hort et al. 2023d).

The rest of the paper is organized as follows. Section 2 provides the background and related work on fairness research, including fairness metrics and bias mitigation methods. Section 3 introduces our approach that is used to adapt trained classification models. The experimental design is described in Section 4. Threats are outlined in Section 4.5, while experiments and results are presented in Section 5. Section 6 concludes.

## Background and Related Work

This section introduces some background on the fairness of software systems, measuring fairness, and bias mitigation methods that have been proposed to improve the fairness of software systems.

### Software Fairness

In recent years, the fairness of software systems has risen in importance, and gained attention from both the software engineering (Zhang et al. 2020; Brun and Meliou 2018; Zhang and Harman 2021; Horkoff 2019; Chakraborty et al. 2020; Hort et al. 2021; Chen et al. 2022b; Sarro 2023; Hort et al. 2023c) and the machine learning research communities (Berk et al. 2017; Kamishima et al. 2012; Kamiran et al. 2012; Calders and Verwer 2010).

While software systems can be designed to reduce discrimination, previous work has observed that this is frequently accompanied by a reduction of the accuracy or correctness of said models (Kamiran and Calders 2012; Feldman et al. 2015; Corbett-Davies et al. 2017; Hort et al. 2023c).

The power of multi-objective approaches can improve such fairness-accuracy trade off (Sarro 2023). Hort et al. (2023c) showed that multi-objective evolutionary search is effective to simultaneously improve for semantic correctness and fairness of word embeddings model. Chen et al. (2022b) proposed MAAT, a novel ensemble approach able to combines ML models optimized for different objectives: fairness and ML performance. Such a combination allow MAAT to outpefrom state-of-the-art methods in 92.2% of the overall cases evaluated. Chakraborty et al. (2020) also integrated bias mitigation into the design of ML software by leveraging a multi-objective search for hyperparameter tuning of a Logistic Regression model. This work has inspired our approach to integrate bias mitigation into the software development process, however at a different stage. While Chakraborty et al. (2020) considered pre- and in-processing approach for bias mitigation, we propose a post-processing approach. Moreover, our approach is not focused on a single classification model, but can be transferred to multiple ones, as we show by using it to improve Logistic Regression and Decision Tree models. Lastly, while their multi-objective optimization does not prevent the improvement of accuracy and fairness at the same time, our approach demands the improvement of both. Perera et al. (2022) proposed a search-based fairness testing approach for testing regression-based machine learning systems, and their empirical results revealed that it is effective to reduce group discrimination in Emergency Department wait-time prediction software.

To ensure fair software, testing methods have been also proposed to address individual discrimination (Horkoff 2019; Zhang et al. 2020; Zhang and Harman 2021; Ma et al. 2022). Tools such as Themis (Galhotra et al. 2017; Angell et al. 2018) and AEQUITAS (Udeshi et al. 2018) are able to generate tests to detect individual discrimination. Similarly, Aggarwal et al. (2019) created tests to detect individual discrimination, however do this in a black-box manner. Ma et al. (2022) proposed a novel an approach for the selection of the initial seeds to generate individual discrimination instances (IDIs) for fairness testing, dubbed I &D, which is effective for improving model fairness. We refer the reader to a comprehensive survey on fairness testing (Chen et al. 2022a).

Empirical studies haven also been carried out by the software engineering community to gain insight on software fairness. Biswas and Rajan (2020) investigated fairness and bias mitigation of real-world crowd-sourced ML models. Furthermore, Harrison et al. (2020) studied the way in which humans perceive the fairness of ML models. Zhang and Harman (2021) found that the fairness of ML software can be improved by using a richer feature set for training. Hort and Sarro (2021) pointed out that reducing the bias of ML software can come at the cost of losing the ability to differentiate between desired features. To allow for a benchmarking of bias mitigation methods, Hort et al. (2021) proposed Fairea which provides a baseline and quantitative evaluation of fairness-accuracy trade-offs. Fairea has been adopted by subsequent studies Chen et al. (2023a) to carry out the most comprehensive empirical study to date of 17 state-of-the-art bias mitigation methods for ML classifiers, evaluated with 11 ML performance metrics, 4 fairness metrics, and 20 types of fairness-performance trade-off assessment, applied to 8 widely-adopted software decision tasks. This study revealed that the bias mitigation methods significantly decrease ML performance in 53% of the studied scenarios (ranging between 42% and 66% according to different ML performance metrics), thus suggesting the need of methods able to improve the accuracy-fairness trade-off. Chen et al. (2024) empirically analysed the effectiveness of 11 state-of-the-art fairness improvement methods when considering multiple protected attributes. They found that improving fairness for a single protected attribute can largely decrease fairness regarding unconsidered protected attributes. Intersectional bias (which encompasses multiple sensitive attributes at the same time) is an open challenge in software fairness (Sarro 2023). We refer the reader to the work by Gohar and Cheng (2023) for a survey on this topic.

### Bias Mitigation Methods

Bias can occur at any stage of the machine learning system development. To repair bias, researchers have applied bias mitigation methods in three different stages: pre-processing, in-processing and post-processing (Friedler et al. 2019; Hort et al. 2023b).

Pre-processing methods aim at processing the training data to reduce bias in the data. Approaches include the reweighing of training data (Kamiran and Calders 2012; Calders et al. 2009), editing of labels and features (Calmon et al. 2017; Feldman et al. 2015), data obfuscation (Zemel et al. 2013), generation of additional data (Chakraborty et al. 2021) and removal of data points (Žliobaite et al. 2011; Chakraborty et al. 2020; Chen et al. 2022b). Pre-processing methods are applied on the training data, which provides the benefit that they can be applied to any classification algorithm. On the other hand, this could lead to uncertainty of results, as they do not take the training algorithms into account.

In-processing methods aim to mitigate bias during training by optimizing the ML algorithms themselves. These include adversarial learning (Zhang et al. 2018), fairness constraints (Kamishima et al. 2012; Calders et al. 2013; Berk et al. 2017), adaptation of split rule for decision trees (Kamiran et al. 2010), decision boundary (un)fairness (Zafar et al. 2017), latent-unbiased variables (Calders and Verwer 2010), hyperparameter tuning (Tizpaz-Niari et al. 2022). gerrymandering (Kearns et al. 2018), and meta algorithms (Celis et al. 2019). While in-processing methods are able to impose specific fairness goals into the training procedure, they are depending on the classification models they are designed for.

Post-processing methods apply changes, once a classification model has been trained. Similar to pre-processing algorithms, post-processing methods can often be applied to any classification algorithm. Moreover, they do not require access to training data or the learning algorithm. Herein we propose a novel post-processing method, therefore in the following we discuss the most common post-processing methods, which are also used as a benchmark in our experiments (Section 5), and the main difference with our proposed approach. We refer the reader to the work by Hort et al. (2023b) for a comprehensive survey on the state-of-the-art bias mitigation methods.

Kamiran et al. (2012, 2018) proposed Reject Option based Classification (ROC), which exploits predictions with high uncertainty. This follows the intuition that discriminatory decisions are made close to the decision boundary and therefore with uncertainty. Given a region with low confidence (e.g., labels close to 0.5 in binary classification), instances belonging to the unprivileged group receive a favorable label, and instances of the privileged group an unfavorable label. Instances outside the low confidence region remain unchanged.

Other than modifying predictions in a post-processing stage, trained classifiers can be addressed as well. Savani et al. (2020) called the post-processing of trained classification models “intra-processing” and proposed an approach for modifying the weights of Neural Networks.

Kamiran et al. (2010) applied leaf relabeling, as a post-processing method on already trained Decision Trees. Usually, labels of leaves are determined by the majority class of the training data which is classified by this particular leaf node. In their debiasing method, leaves are relabeled to reduce discrimination (e.g., a leaf that is returning “false” is changed to return “true”), while also keeping the loss in accuracy minimal. In particular, each leaf node is investigated to select and relabel the leaf with the highest ratio of discrimination reduction and accuracy loss. Their approach assumes that, in order to lower discrimination of DTs, one has to lower accuracy.

Hardt et al. (2016) proposed a post-processing method based on equalized odds. A classifier is said to satisfy equalized odds when it is independent of protected attribute and true label (i.e., true positive and false positive rates across privileged and unprivileged group are equal). Given a trained classification model, they used linear programming to derive an unbiased one. Another variant of the equalized odds bias mitigation method has been proposed by Pleiss et al. (2017). In contrast to the original equalized odds method, they used calibrated probability estimates of the classification model (e.g., if 100 instances receive $p=0.6$, then 60% of them should belong to the favorable label 1).

Our herein proposed post-processing approach differs from the leaf relabeling approach proposed by Kamiran et al. (2010), as we do apply changes to the classification model only if they increase accuracy and reduce bias. In other words, our approach is the first to deliberately optimize classification models for accuracy and fairness at the same time, unlike existing methods that are willing to reduce bias at the cost of accuracy (Berk et al. 2017). Overall, we apply a search procedure rather than deterministic approaches (Kamiran et al. 2010, 2012, 2018; Hardt et al. 2016; Pleiss et al. 2017) and we do not assume that bias reduction has to come with a decrease in accuracy. To the best of our knowledge our proposal is the first to improve classification models according to *both* fairness and accuracy by mutating the classification model itself, rather than manipulating the training data or the predictions.

### Fairness Measurement

There are two primary methods to measure fairness of classification models: individual fairness and group fairness (Speicher et al. 2018). While *individual fairness* is concerned with an equal treatment of similar individuals (Dwork et al. 2012), *group fairness* requires equal treatment of different population groups. Such groups are divided by protected attributes, such as race, age or gender. Thereby, one group is said to be *privileged* if it is more likely to get an advantageous outcome than another, *unprivileged* group.

Due to the difficulty of determining the degree of similarity between individuals (Jacobs and Wallach 2021), it is common in the literature to focus on group fairness metrics. In particular, we investigate three group fairness metrics (all publicly available in the AIF360 framework (Bellamy et al. 2018)) to measure the fairness of a classification model, which are frequently used in the domain of software fairness (Zhang and Harman 2021; Chakraborty et al. 2020, 2021; Hort et al. 2021) and are usually optimized by existing bias mitigation methods such as Statistical Parity Difference, Average Odds Difference, and Equal Opportunity Difference.

Proceeding, we use $\hat{y}$ to denote a prediction of a classification model. We use *D* to denote a group (privileged or unprivileged). We use *Pr* to denote probability.

The Statistical Parity Difference (SPD) requires that predictions are made independently of protected attributes (Zafar et al. 2017). Therefore, favourable and unfavourable classifications for each demographic group should be identical over the whole population (Dwork et al. 2012):

1 $$\begin{aligned} SPD= Pr(\hat{y} = 1 | D = unprivileged)\nonumber \\ - Pr(\hat{y} = 1 | D = privileged) \end{aligned}$$

The Average Odds Difference (AOD) averages the differences in False Positive Rate (FPR) and True Positive Rate (TPR) among privileged and unprivileged groups (Hardt et al. 2016):

2 $$\begin{aligned} AOD= \frac{1}{2}((FPR_{D=unprivileged} - FPR_{D=privileged})\nonumber \\ + (TPR_{D=unprivileged} - TPR_{D=privileged})) \end{aligned}$$

The Equal Opportunity Difference (EOD) corresponds to the TPR difference (Hardt et al. 2016):

3 $$\begin{aligned} EOD= TPR_{D=unprivileged} - TPR_{D=privileged} \end{aligned}$$

Following previous work on fairness in SE (Chakraborty et al. 2020; Zhang and Harman 2021), we are interested in the absolute values of these metrics. Thereby, each metric is minimized at zero, indicating that no bias is residing in a classification model.

## Proposed Approach

This section introduces the search-based procedure we propose for mutating classification models to simultaneously improve both accuracy and fairness. In addition, we describe implementation details for two classification models (Logistic Regression, Decision Trees) to perform such a procedure.

### Procedure

Our search-based post-processing procedure aims to iteratively mutate a trained classification model in order to improve both accuracy and fairness at the same time. For this purpose, we require a representation of the classification model that allows changes (“mutation”) to the prediction function. To simplify the mutation process, we apply mutation incrementally (i.e., repeatedly changing small aspects of the classifier). Such a procedure is comparable to the local optimisation algorithm hill climbing. Based on an original solution, hill climbing evaluates neighboring solutions and selects them only if it improves the original fitness (Harman et al. 2010). We mutate a trained classification model *clf* with the goal to achieve improvements in accuracy and fairness. In this context, the fitness function measures the accuracy and fairness of *clf* on a validation dataset (i.e., a dataset that has not been used during the initial training of *clf*). “Accuracy” (acc) refers to the standard accuracy in machine learning, which is the number of correct predictions against the total number of predictions. To measure fairness, we use the three fairness metrics introduced in Section 2.3 (SPD, AOD, EOD).

Algorithm 1 outlines our procedure to improve accuracy and fairness of a trained classification model *clf*. In line 4, *fitness*(*clf*) determines the fitness of the modified classification model in terms of accuracy ($acc'$) and a fairness metric ($fair'$). In our empirical study we experiment with three different fairness metrics (see Section 2.3), one at a time. If desired, *fitness*(*clf*) can also be modified to take multiple fairness metrics into account simultaneously.

We only apply a mutation if the accuracy and fairness of the mutated model ($acc',fair'$) are better than the accuracy and fairness of the previous classification model (*acc*, *fair*) (Line 5). If that is not the case, the mutation is reverted ($undo\_mutation$) and the procedure continues until the terminal condition is met. A mutation of the trained model at each iteration of the search process that leads to an improvement in one objective (either accuracy or fairness) will almost certainly change the other objective at the same time. If the other objective is not worsened, the change is kept; otherwise, the change is reverted. This effect is accumulated over each iteration.

To show the generalizability of the approach, and in line with previous work (Kamiran et al. 2012, 2018; Chakraborty et al. 2020), we use the default configuration, as provided by scikit (Pedregosa et al. 2011) to train the classification models before applying our post-processing procedure.

**Algorithm 1:**
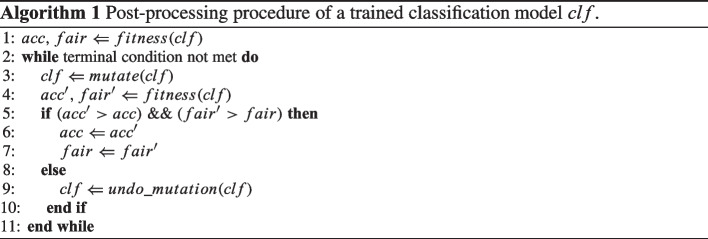
Post-processing procedure of a trained classification model *clf*.

### Logistic Regression

**Representation.** Logistic Regression (LR) is a linear classifier that can be used for binary classification. Given training data, LR determines the best weights for its coefficients. Below, we illustrate the computation of the LR prediction with four tuneable weights ($b_0,b_1,b_2,b_3$). At first, Equation 4 presents the computation of predictions with a regular linear regression classifier. To make a prediction, LR uses this the *Linear* prediction in a sigmoid function (Equation 5):

4 $$\begin{aligned} Linear(x_1,x_2,x_3) = b_0 + b_1 x_1 + b_2 x_2 + b_3 x_3 \end{aligned}$$

5 $$\begin{aligned} P(Y) = \frac{1}{1+e^{-Y}} \end{aligned}$$

This *prediction* function determines the binary label of a 3-dimensional input ($x_1,x_2,x_3$). In a binary classification scenario, we treat predictions $\ge 0.5$ as label 1, and 0 otherwise.

This shows that the binary classification is determined by *n* variables ($b_0 \dots b_{n-1}$). To represent an LR model, we store the *n* coefficients in an n-dimensional vector.

#### Mutation

Given that an LR classification model can be represented by one-dimensional vector, we mutate single vector elements to create mutated variants of the model. In particular, we pick an element at random and multiply it by a value within a range of $\{-10\%, 10\%\}$. We performed an analysis on different degrees of noise and mutation operators for LR models in Section 5.4.

### Decision Tree

**Representation.** Decision Trees (DT) are classification models that solve the classification process by creating tree-like solutions, which create leaves and branches based on features of the training data. We are interested in binary DTs. In binary DTs, every interior node (i.e., all nodes except for leaves) have exactly two child nodes (left and right).

#### Mutation

We use pruning as a means to mutate DTs. The pruning process deletes all the children of an interior node, transforming it into a leaf node, and has shown to improve the accuracy of DT classification in previous work (Breiman et al. 1984; Quinlan 1987; Breslow and Aha 1997). In particular, we pick an interior node *i* at random and treat it as a leaf node by removing all subjacent child nodes. We choose to use pruning, instead of leaf relabeling, because preliminary experiments showed that pruning outperforms leaf relabeling (i.e., Kamiran et al. (2010) used leaf relabeling in combination with an in-processing method but not in isolation).

## Experimental Setup

In this section, we describe the experimental design we carry out to assess our search-based bias repair method for binary classification models (i.e., Logistic Regression and Decision Trees). We first introduce the research questions, followed by the subjects and the experimental procedure used to answer these questions.

### Research Questions

Our evaluation aims to answer the following research questions:

**RQ1: To what extent can the proposed search-based approach be used to improve both, accuracy and fairness, of binary classification models?**

To answer this question, we apply our post-processing approach to LR and DTs (Section 3) on four datasets with a total of six protected attributes (Section 4.2).

The search procedure is guided by accuracy and each of the three fairness metrics (SPD, AOD, EOD) separately. Therefore, for each classification model, we perform 3 (fairness metrics) x 6 (datasets) = 18 experiments. For each of the fairness metrics, we mutate the classification models and measure changes in accuracy and the particular fairness metric used to guide the search (e.g., we post-process LR based on accuracy and SPD). We then determine whether the improvement in accuracy and fairness (as explained in Section 3) achieved by mutating the classification models are statistically significant, in comparison to the performance of the default classification model.

Furthermore, we compare optimization results from post-processing with existing bias mitigation methods:

**RQ2: How does the proposed search-based approach compare to existing bias mitigation methods?**

We address this research question in two steps. First, we perform a comparison with post-processing bias mitigation methods, which are applied at the same stage of the development process as our approach (RQ2.1). Afterwards, we compare our post-processing approach to pre- and in-processing methods (RQ2.2).

To answer both questions (RQ2.1 and RQ2.2), we benchmark our approach against existing and widely-used bias mitigation methods: three post-processing methods, three pre-processing methods and one in-processing method, which are all publicly available in the AIF360 framework (Bellamy et al. 2018). In particular, we applied these existing bias mitigation methods to LR and DTs on the same set of problems (i.e., the four datasets used also for RQ1 and RQ3) in order to compare their fairness-accuracy trade-off with the one achieved by our proposed approach. A description of the benchmarking bias mitigation methods is provided in Section 4.3, whereas the datasets used are described in Section 4.2.

While the objectives considered during the optimization procedure are improved, this has shown to carry detrimental effects on other objectives (Ferrucci et al. 2010; Chakraborty et al. 2020). Therefore, we determine the impact optimization for one fairness metric has on the other two fairness metrics, which have not been considered during the optimization procedure:

**RQ3: What is the impact of post-processing guided by a single fairness metric on other fairness metrics?**

To answer this question, we apply our post-processing method on LR and DTs. While optimizing for each of the three fairness metrics, we measure changes of the other two. We are then able to compare the fairness metrics before and after the optimization process, and visualize changes using boxplots. Moreover, we can determine whether there are statistically significant changes to “untouched” fairness metrics, which are not optimized for.

We perform additional experiments to gain insights on the importance of parameters when applying our post-processing method (i.e., terminal condition and mutation operations), and the performance of advanced binary classification models (e.g., neural networks) in comparison to Logistic Regression and Decision Tree classifiers. The investigation of parameter choices is addressed in Section 5.4, advanced classification models are investigated in Section 5.5.

### Datasets

We perform our experiments on four real-world datasets used in previous software fairness work (Chakraborty et al. 2020; Zhang and Harman 2021) with a total of six protected attributes.

The Adult Census Income (**Adult**) (Kohav 2023) contains financial and demographic information about individuals from the 1994 U.S. census. The privileged and unprivileged groups are distinguished by whether their income is above 50 thousand dollars a year.

The Bank Marketing (**Bank**) (Moro et al. 2014) dataset contains details of a direct marketing campaign performed by a Portuguese banking institution. Predictions are made to determine whether potential clients are likely to subscribe to a term deposit after receiving a phone call. The dataset also includes information on the education and type of job of individuals.

The Correctional Offender Management Profiling for Alternative Sanctions (**COMPAS**) (propublica 2023) dataset contains the criminal history and demographic information of offenders in Broward County, Florida. To indicate whether a previous offender is likely to re-offend, they receive a *recidivism* label.

The Medical Expenditure Panel Survey (**MEPS19**) represents a large scale survey of families and individuals, their medical providers, and employers across the United States.^3^ The favourable label is determined by “Utilization” (i.e., how frequently individuals frequented medical providers).

In Table 1, we provide the following information about the four datasets: number of rows and features, the favourable label and majority class. In addition, we list the protected attributes for each dataset (as provided by the AIF360 framework (Bellamy et al. 2018)), which are investigated in our experiments, and the respective privileged and unprivileged groups for each protected attribute.

**Table 1:** Datasets used in our empirical study

Dataset	Size	Attributes	Favourable Label	Majority Label	Protected	Privileged - Unprivileged
Adult	48,842	14	1 (income $>50k$)	0 (75%)	Sex	Male - female
					Race	White - non white
COMPAS	7,214	28	0 (No recid)	0 (54%)	Sex	Female - male
					Race	Caucasian - not Caucasian
Bank	41,188	20	1 (yes)	0 (87%)	Age	$\ge 25$ - $<25$
MEPS19	15,830	138	1 ($\ge 10$ visits)	0 (83%)	Race	White - non-white

### Benchmark Bias Mitigation Methods

As our proposed method belongs to the category of post-processing methods, we compare it with all the state-of-art post-processing bias mitigation methods made publicly available in the AIF360 framework (Bellamy et al. 2018), as follows (Section 2.2):

- Reject Option Classification (ROC) (Kamiran et al. 2012, 2018);
- Equalized odds (EO) (Hardt et al. 2016);
- Calibrated Equalized Odds (CO) (Pleiss et al. 2017).

AIF360 (Bellamy et al. 2018) provides ROC and CO with the choice of three different fairness metrics to guide the bias mitigation procedure (Section 2.3). ROC can be applied with SPD, AOD, and EOD. CO can be applied with False Negative rate (FNR), False Positive Rate (FPR), and a “weighed” combination of both. We apply both, ROC and CO, with each of the available fairness metrics. EO does not provide choices for fairness metrics to users.

While our focus lies on the empirical evaluation of our post-processing approach with approaches of the same type, we also consider a comparison with pre- and in-processing methods (RQ2-2, Section 5.5). In particular, we compare our approach to the following pre-processing and in-processing methods:

- Optimized Pre-processing (OP) (Calmon et al. 2017): Probabilistic transformation of features and labels in the dataset.
- Learning Fair Representation (LFR) (Zemel et al. 2013): Intermediate representation learning to obfuscate protected attributes.
- Reweighing (RW) (Kamiran and Calders 2012; Calders et al. 2009): Reweighing the importance (weigh) of instances from the privileged and unprivileged group in the dataset.
- Exponentiated gradient reduction (RED) (Agarwal et al. 2018): Two player game to find the best randomized classifier under fairness constraints.

The three pre-processing methods (OP, LFR, RW) are classification model-agnostic and can be easily be applied Logistic Regression and Decision Tree models (i.e., training data can be changed independent of the classification model used). Whereas, in order to apply RED, the in-processing approach proposed by Agarwal et al. (2018), one needs to provide a classification model (Logistic Regression or Decision Tree) and a fairness notion. In our case, we apply RED with three different fairness notions: “DemographicParity” ($RED_{DP}$), “EqualizedOdds” ($RED_{EO}$), “TruePositiveRate” ($RED_{TPR}$). These three notions coincide with our evaluation metrics, SPD, AOD and EOD, respectively.

**Fig. 1:**
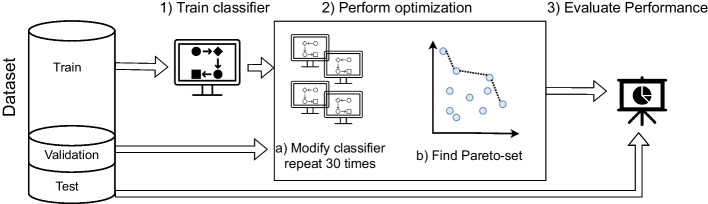
Empirical evaluation of a single data split

### Validation and Evaluation Criteria

To validate the effectiveness of our post-processing approach to improve accuracy and fairness of binary classification models, we apply it to LR and DT. Since our optimization approach applies random mutations, we expect variation in the results. Figure 1 illustrates the empirical evaluation procedure of our method for a single datasplit. At first, we split the data in three sets: training (70%), validation (15%), test (15%).^4^ To mitigate variation, we apply each bias mitigation method, including our newly proposed approach on 50 different data splits.

The training data is used to create a classifier which we can post-process. Once a classifier is trained (i.e., Logistic Regression or Decision Tree), we apply our optimization approach 30 times (Step 2).^5^ To then determine the performance (accuracy and fairness) of our approach on a single data split, we compute the Pareto-optimal set^6^ based on the performance on the validation set. Once we obtain the Pareto-set of optimized classification models based on their performance on the validation set, we average their performance on the test set. Performance on the test set (i.e., accuracy and fairness) is used to compare different bias mitigation methods and determine their effectiveness. Each run of our optimization approach is limited to 2, 500 iterations (terminal condition, Algorithm 1). The existing post-processing methods are deterministic, and therefore applied only once for each data split.

To assess the effectiveness of our approach (RQ1) and compare it with existing bias mitigation methods (RQ2), we consider both summary statistics (i.e., average accuracy and fairness), statistical significance tests and effect size measures, and Pareto-optimality. Furthermore, we use boxplots to visualize the impact of optimizing accuracy and one fairness metric on the other two fairness metrics (RQ3).

*Pareto-optimality* states that a solution *a* is not worse in all objectives than another solution *b* and better in at least one (Harman et al. 2010). We use Pareto-optimality to both measure how often our approach dominates the default classification model or is Pareto-optimal, and to plot the set of solutions found to be non-dominated (and therefore equally viable) with respect to the state-of-the-art (RQs1-2). In the case where there are two objectives, such as ours, this leads to a two dimensional Pareto surface.

To determine whether the differences in the results achieved by all approaches are statistical significant, we use the Wilcoxon Signed-Rank test, which is a non-parametric test that makes no assumptions about underlying data distribution (Wilcoxon 1992). We set the confidence limit, $\alpha$, at 0.05 and applied the Bonferroni correction for multiple hypotheses testing ($\alpha /K$, where *K* is the number of hypotheses).^7^ This correction is the most conservative of all corrections and its usage allows us to avoid the risk of Type I errors (i.e., incorrectly rejecting the Null Hypothesis and claiming predictability without strong evidence). In particular, depending on the RQ, we test the following null hypothesis:

(RQ1) $H_0$: *The fairness and accuracy achieved by* $approach_x$ *is not improved with respect to the default classification model*. The alternative hypothesis is as follows: $H_1$: *The fairness and accuracy achieved by* $approach_x$ *improves with respect to the default classification model*. In this context, “improved” means that the accuracy is increased and fairness metric values are decreased (e.g., a SPD of 0 indicates that there is no unequal treatment of privileged and unprivileged groups).

(RQ3) $H_0$: *Optimizing for accuracy and fairness metric* $m_1$ *does not improve fairness metric*$m_2$ *with respect to the default classification model*. The alternative hypothesis is as follows: $H_1$: *Optimizing for accuracy and fairness metric* $m_1$ *improves fairness metric*$m_2$ with *respect to the default classification model*. For this RQ, we summarise the results of the Wilcoxon tests by counting the number of win-tie-loss as follows: p–value<0.01 (win), p–value>0.99 (loss), and 0.01$\le$ p–value $\ge$0.99 (tie), as done in previous work (Sarro et al. 2017; Kocaguneli et al. 2011; Sarro et al. 2018; Sarro and Petrozziello 2018).

In addition to evaluating statistical significance, we measure the effect size based on the Vargha and Delaney’s $\hat{A}_{12}$ non-parametric measure (Vargha and Delaney 2000), which does not require that the data is normally distributed (Arcuri and Briand 2014). The $\hat{A}_{12}$ measure compares an algorithm *A* with another algorithm *B*, to determine the probability that *A* performs better than *B* with respect to a performance measure *M*:

6 $$\begin{aligned} \hat{A}_{12} = (R_1/m - (m + 1)/2)/n \end{aligned}$$

In this formula, *m* and *n* represent the number of observations made with algorithm *A* and *B* respectively; $R_1$ denotes the rank sum of observations made with *A*. If *A* performs better than *B*, $\hat{A}_{12}$ can display one of the following effect sizes: $\hat{A}_{12} \ge 0.72$ (large), 0.64 $< \hat{A}_{12} < 0.72$ (medium), 0.56 $< \hat{A}_{12} < 0.64$ (small), although these thresholds are not definitive (Sarro et al. 2016).

### Threats to Validity

The *internal* validity of our study relies in the confidence that the experimental results we obtained are trustworthy and correct. To alleviate possible threats to the internal validity, we applied our post-processing method and existing bias mitigation methods 50 times, under different train/validation/test splits. This allowed us to use statistical significance tests to further assess our results and findings. We have used traditional measures used in the software fairness literature to assess ML accuracy, while we recognise alternative measures could be used to take into account data imbalance (Chen et al. 2023b; Moussa and Sarro 2022).

Threats to *external* validity related to generalizability of our results, are primarily concerned with the datasets, approaches and metrics we investigated. To mitigate this threat we have considered in this study all datasets publicly available which have been previously used in the literature to solve the same problem. Using more data in the future will further increase the generalizability of our results. Furthermore, we have successfully applied our post-processing method on two inherently different classification models (Logistic Regression, Decision Trees), which strengthens the confidence that our approach could be applied to other binary classifiers. We have also explored all state-of-the-art post-processing debiasing methods in addition to three pre-processing and one in-processing method available from the AIF360 framework (Bellamy et al. 2018) (version 0.3.0), which is publicly available, to strengthen the generalizability and reproducibility of our work.

To mitigate possible threats to *construct* validity, and support the applicability and generalizability of our approach, and allow for the replication and extension of our work, we have made our scripts and results publicly available (Hort et al. 2023d).

## Results

This section presents the results of our experiments to answer the research questions explained in Section 4.1.

**Table 2:** RQ1-Logistic Regression: Average accuracy and fairness of non-dominated solutions over 50 different data splits (i.e., for each data split, we select the non-dominated solutions and average their performance on the test set)

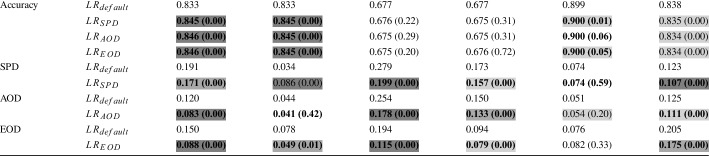	

**Bold** values indicate improvements over the default classification model. The p-value of the Wilcoxon Signed-Rank test comparing each approach with the default Logistic Regression model, is given in brackets for each metric. Colors are used to show the effect size (  ,  ,

**Table 3:** RQ1-Decision Tree: Average accuracy and fairness of non-dominated solutions over 50 different data splits (i.e., for each data split, we select the non-dominated solutions and average their performance on the test set)

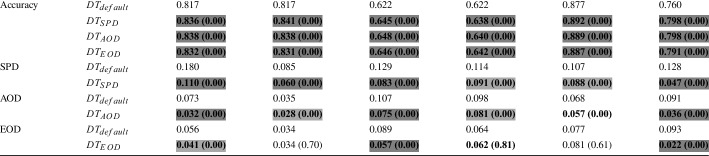	

**Bold** values indicate improvements over the default classification model. The p-value of the Wilcoxon Signed-Rank test comparing each approach with the default Decision Tree model, is given in brackets for each metric. Colors are used to show the effect size (  ,  ,

### RQ1. Fairness-Accuracy Improvement

In the first research question, we investigate whether our post-processing approach is able to improve both fairness and accuracy when applied to binary classification models (namely LR and DT). The baseline considered is the default classification model. We apply our approach on four datasets, as outlined in Section 4.4. In total, we apply post-processing with three different configurations, to optimize for accuracy and one of the three fairness metric at a time. We will call those configurations $DT_{SPD}$, $DT_{AOD}$, $DT_{EOD}$, $LR_{SPD}$, $LR_{AOD}$, $LR_{EOD}$ to determine the classification model and the fairness metric considered during optimization. These configurations are applied to four datasets on 50 train/validation/test splits and repeated 30 times. Tables 2 and 3 show these results for Logistic Regression and Decision Trees respectively. These tables show the results of the default classification model and the three optimization configurations.

We can see that our post-processing approach is able to improve the accuracy of the two classification models (LR and DT) in 27 out of 36 cases. In the half of the cases the accuracy of LR is statistically significant better (6 out of 18 cases) or comparable (3 out of 18 cases) with respect to the default model, while in 6 out of 18 cases it is reduced although no statistical significant difference is observed. In the remaining three cases, all on the MEPS19 datasets, accuracy is statistically worse with a small effect size.

All the 18 out of 18 cases improve the accuracy of DT, all of which are statistically significant with large effect sizes.

When investigating the impact of our post-processing approach on each of the three fairness metrics (i.e., mutation is applied if the particular fairness metric and accuracy are improved), we compare the fairness of the default classification model with the configuration to optimize for that particular metric (e.g., we compare the SPD of the default LR with the SPD achieved by $LR_{SPD}$). Therefore, instead of 18 cases for LR and DT, we have six comparisons for each metric.

For each of the three fairness metrics (SPD, AOD, EOD) our post-processing approach is able to improve fairness on 5 out of 6 datasets on LR. $LR_{SPD}$ is not able to achieve SPD improvements on the Adult dataset (protected attribute = “race”), $LR_{AOD}$ and $LR_{EOD}$ are not able to achieve fairness improvements on the Bank dataset. Among the 15 out of 18 cases that improve fairness on LR, 11 are statistically significant, with six of those having large effect sizes. Furthermore, it can be noted that the instances where our approach is not able to improve fairness, already have a low bias score. According to the online tool of the AIF360 framework (Bellamy et al. 2018), values $\le 0.1$ can be seen as fair, when investigating SPD, AOD and EOD.^8^ Applied to DTs, our post-processing approach improves fairness for 16 out of 18 cases. In particular, in 6 out of 6 cases $DT_{SPD}$ and $DT_{AOD}$ achieve statistically significant fairness improvements on their respective fairness metric. In 3 out of 6 cases, $DT_{EOD}$ achieves statistically significant improvements. In the remaining two cases (i.e., EOD on the Adult-race and Bank-Age datasets), our approach is not able to significantly improve fairness, likely because the default model already shows a low bias ($\le 0.1$).

Overall, the three post-processing configurations achieve improvements in both accuracy and fairness in 22 out of 36 cases, and improvements in at least one of the two (i.e., either accuracy and fairness) in the remaining 14 out of 36 cases. Notably, our post-processing approach improves accuracy and fairness of DTs in 16 out of 18 cases.

In addition to comparing the average performance of our optimization approach for each data-split (i.e., we average accuracy and fairness of all solutions in the Pareto-front), we perform a comparison of each solution in the Pareto-front with the default classification model. Table 4 shows the results. For each combination of datasets and metric optimized by our approach, we compute the percentage of solutions that: dominate the default model, are Pareto-optimal, are dominated by the default model. This comparison (e.g., do solutions in the Pareto-front dominate the default classification model?) is performed for each data-split and weighted accordingly, such that each data-split has the same contribution to the results (e.g., a data-split with 10 solutions in the Pareto-front is treated equally as a data-split with 2 solutions in the Pareto-front). Our post-processing methods applied on Logistic Regression achieves comparable or better performance than the default model in 91% of the cases across all datasets studied, and, specifically, it dominates the default model in 38% of the cases and is dominated in only 9% of the cases. This shows that our approach is a useful tool for optimizing LR models (i.e., developers are either able to choose a strictly better model, or models with competitive fairness-accuracy trade-offs). When we apply our approach to DTs, we observe an even higher performance improvement: It dominates the default DT models in 78% of the cases and not dominated in the remaining cases.

**Table 4:** RQ1: Comparison of each individual run of our approach (30 runs over 50 datasplits) against the default classification model

		Adult		Compas		Bank	Meps19	
		Sex	Race	Sex	Race	Age	Race	$\Sigma$
LR	SPD	59-41-0	0-98-2	36-57-7	38-47-16	37-50-14	25-68-8	32-60-8
	AOD	65-34-1	50-50-0	36-54-10	37-48-16	26-50-24	15-65-19	38-50-12
	EOD	71-29-0	61-39-0	37-58-6	41-44-15	31-49-19	17-72-11	43-48-8
$\Sigma$		65-35-0	37-62-1	36-56-7	39-46-16	31-50-19	19-68-13	38-53-9
DT	SPD	100-0-0	100-0-0	91-9-0	76-23-2	69-31-0	99-1-0	89-11-0
	AOD	100-1-0	71-29-0	85-14-1	69-31-1	63-37-0	95-5-0	80-19-0
	EOD	78-22-0	54-46-0	78-20-2	47-52-1	43-57-0	89-11-0	65-35-0
$\Sigma$		92-8-0	75-25-0	85-15-1	64-35-1	58-42-0	94-6-0	78-22-0

For each dataset and metric, we measure the percentage of runs that: dominate the default model - are Pareto-optimal - are dominated by the default model

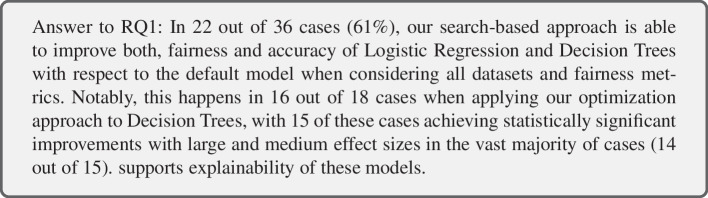



**Fig. 2:**
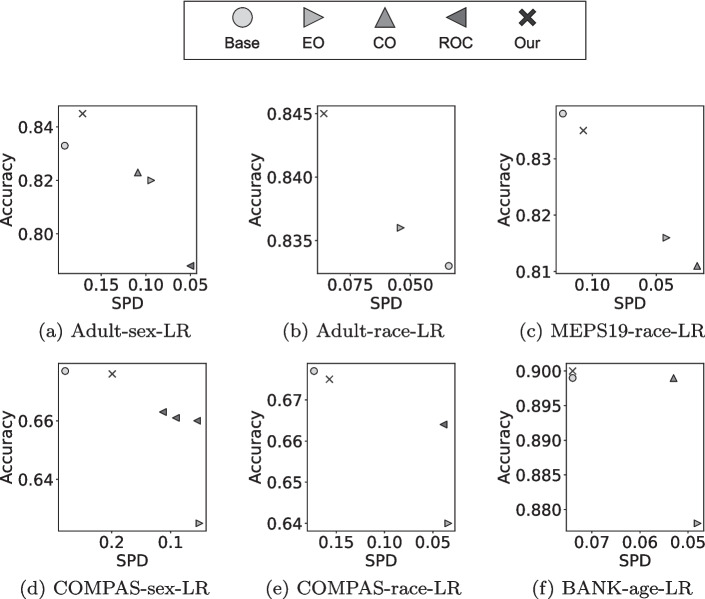
RQ2: Comparison of our proposed approach against existing bias mitigation methods and default classification models based on Pareto-optimality. The figure shows six exemplary comparisons for LR and SPD

### RQ2. Comparison to Existing Bias Mitigation Methods

#### RQ2-1. Comparison to Post-Processing Methods

To answer RQ2.1, we compare our post-processing method against three existing post-processing bias mitigation methods (Section 4.3) applied to LR and DT on the same datasets (Adult, COMPAS, Bank, MEPS19) by using identical train/validation/test splits, as described in Section 4. The mean performance of these methods over 50 data splits, and of our post-processing method, are shown in Figure 2. While Figure 2 only includes six cases for LR and measuring SPD, the remaining results for other metrics and DTs are available in our online appendix (Hort et al. 2023d). In each sub-figure, we show the performance of every non-dominated bias mitigation method on the respective dataset and fairness metric. A summary on how often each bias mitigation method is part of the Pareto-front is provided in Table 5.

When comparing the accuracy of classification models achieved after applying our post-processing method against the existing bias mitigation methods, we observe that all of the existing bias mitigation methods have a lower accuracy. Moreover, all of the existing bias mitigation methods reduce the accuracy of the default classification model, thereby conforming to the fairness-accuracy trade-off. On the other end, our approach, which takes into account accuracy in the bias mitigation process, is always able to generate a widely applicable solution (i.e., our approach always produces at least a solution belonging to each of 36 Pareto-fronts, and therefore is never dominated by any of the existing methods).

**Table 5:** RQ2: Frequency of bias mitigation methods in the Pareto-front

	Logistic Regression				Decision Tree			
	Our	CO	ROC	EO	Our	CO	ROC	EO
SPD	6	3	3	6	6	0	2	0
AOD	6	2	2	6	6	0	2	0
EOD	6	2	4	5	6	0	2	1
$\Sigma$	18/18	7/18	9/18	17/18	18/18	0/18	6/18	1/18

Each combination of bias mitigation method and fairness metric is evaluated on six datasets

We can observe a difference in performance of our approach when applied to LR and DT. While our approach, applied to LR, is able to outperform some of the existing bias mitigation methods on the three fairness metrics (CO and ROC), it is only able to dominate EO in 1 out of 18 cases (Bank-age EOD). In the remaining 17 cases, EO has a lower accuracy than our approach while improving fairness to a higher degree. On the other end, when applying our post-processing approach to DTs, it not only produces solutions that dominate the default classification model (as seen in RQ1), but also all investigated bias mitigation methods in 12 out of 18 cases. Furthermore, for DT, our approach outperforms existing bias mitigation methods on the three fairness metrics, in addition to achieving the highest accuracy. In particular, our approach achieves the lowest bias on all three fairness metrics for the Adult, Bank and MEPS19 datasets. Only ROC is able to achieve a lower level of bias for the COMPAS dataset in 6 out of 6 cases, and EO in 1 out of 6 cases. This may be due to the fact that COMPAS is the smallest of the datasets we investigate herein. 
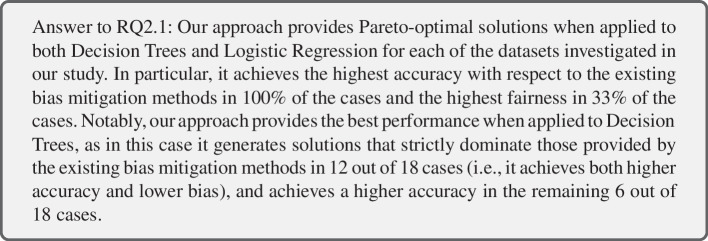

**Table 6:** RQ2-2: Performance comparison with pre-processing (LFR, OP, RW) and in-processing (RED) methods for Logistic Regression

		Adult		Compas		Bank	Meps19
		Sex	Race	Sex	Race	Age	Race
ACC	$LR_{default}$	0.833	0.833	**0.677**	**0.677**	0.899	**0.838**
	$LR_{SPD}$	0.845	**0.845**	0.676	0.675	**0.900**	0.835
	$LR_{AOD}$	**0.846**	**0.845**	0.675	0.675	**0.900**	0.834
	$LR_{EOD}$	**0.846**	**0.845**	0.675	0.676	**0.900**	0.834
	LFR	0.773	0.770	0.549	0.549	0.878	0.795
	OP	0.794	0.803	0.665	0.659		
	REW	0.789	0.803	0.661	0.656	**0.900**	0.835
	$RED_{DP}$	0.783	0.802	0.658	0.651	0.899	0.826
	$RED_{EO}$	0.789	0.803	0.655	0.643	0.897	0.834
	$RED_{TPR}$	0.789	0.803	0.658	0.652	0.899	0.833
SPD	$LR_{default}$	0.191	0.034	0.279	0.173	0.074	0.123
	$LR_{SPD}$	0.171	0.086	0.199	0.157	0.074	0.107
	LFR	0.111	0.069	0.063	0.075	0.032	0.036
	OP	0.115	0.047	0.159	0.124		
	REW	0.066	0.041	0.097	0.060	0.031	0.055
	$RED_{DP}$	**0.017**	**0.014**	**0.043**	**0.038**	**0.023**	**0.019**
AOD	$LR_{default}$	0.120	0.044	0.254	0.150	0.051	0.125
	$LR_{AOD}$	0.083	0.041	0.178	0.133	0.054	0.111
	LFR	0.115	0.087	0.065	0.076	0.052	0.037
	OP	0.094	0.025	0.126	0.096		
	REW	**0.014**	**0.022**	0.087	0.053	**0.043**	**0.029**
	$RED_{EO}$	0.019	0.025	**0.061**	**0.044**	0.050	0.032
EOD	$LR_{default}$	0.150	0.078	0.194	0.094	0.076	0.205
	$LR_{EOD}$	0.088	0.049	0.115	0.079	0.082	0.175
	LFR	0.171	0.137	0.057	0.065	0.084	0.057
	OP	0.151	0.036	0.082	0.072		
	REW	**0.021**	**0.033**	**0.054**	**0.043**	**0.073**	**0.045**
	$RED_{TPR}$	0.033	0.042	0.063	0.049	0.075	0.059

Bold values highlight the best metric values for each dataset

**Table 7:** RQ2-2: Performance comparison with pre-processing (LFR, OP, RW) and in-processing (RED) methods for Decision Trees

		Adult		Compas		Bank	Meps19
		Sex	Race	Sex	Race	Age	Race
ACC	$DT_{default}$	0.817	0.817	0.622	0.622	0.877	0.760
	$DT_{SPD}$	0.836	**0.841**	0.645	0.638	**0.892**	**0.798**
	$DT_{AOD}$	**0.838**	0.838	0.648	0.640	0.889	**0.798**
	$DT_{EOD}$	0.832	0.831	0.646	0.642	0.887	0.791
	LFR	0.747	0.745	0.569	0.571	0.829	0.738
	OP	0.786	0.799	0.658	**0.655**		
	REW	0.787	0.801	0.658	0.652	0.879	0.760
	$RED_{DP}$	0.784	0.801	0.656	0.648	0.877	0.764
	$RED_{EO}$	0.790	0.802	0.658	0.647	0.876	0.758
	$RED_{TPR}$	0.790	0.802	**0.659**	0.650	0.878	0.759
SPD	$DT_{default}$	0.180	0.085	0.129	0.114	0.107	0.128
	$DT_{SPD}$	0.110	0.060	0.083	0.091	0.088	0.047
	LFR	0.167	0.075	0.096	0.066	0.073	0.100
	OP	0.068	0.023	0.104	0.136		
	REW	0.056	**0.014**	0.071	0.091	0.104	0.102
	$RED_{DP}$	**0.018**	**0.014**	**0.040**	**0.038**	**0.027**	**0.037**
AOD	$DT_{default}$	0.073	0.035	0.107	0.098	0.068	0.091
	$DT_{AOD}$	0.032	0.028	0.075	0.081	**0.057**	**0.036**
	LFR	0.137	0.087	0.093	0.067	0.083	0.087
	OP	0.050	0.042	0.087	0.108		
	REW	0.032	0.048	0.070	0.081	0.068	0.068
	$RED_{EO}$	**0.020**	**0.023**	**0.056**	**0.048**	0.070	0.087
EOD	DT-default	0.056	**0.034**	0.089	0.064	**0.077**	0.093
	$DT_{EOD}$	0.041	**0.034**	0.057	0.062	0.081	**0.022**
	LFR	0.170	0.140	0.074	0.053	0.097	0.098
	OP	0.081	0.066	0.058	0.085		
	REW	0.049	0.078	0.061	0.070	**0.077**	0.070
	$RED_{TPR}$	**0.032**	0.039	**0.039**	**0.042**	0.083	0.089

Bold values highlight the best metric values for each dataset

#### RQ2-2. Comparison to Pre- and In-Processing Methods

To answer RQ2-2, we compare our post-processing approach with available pre- and in-processing bias mitigation methods. In particular, we use three pre-processing methods (LFR, OP, REW) and one in-processing method (RED), under consideration of three fairness metrics ($RED_{DP}$, $RED_{EO}$, $RED_{TPR}$), for comparison. Table 6 shows the performance of the bias mitigation methods when applied to Logistic Regression models and Table 7 shows results for Decision Trees. Due to the dimensionality of data (number of features and instances in the dataset), OP could not be applied to the Bank and Meps19 datasets.

The lowest bias for LR models is achieved by REW (pre-processing) and RED (in-processing), while the highest accuracy is achieved by our post-processing approach and the original LR model (Table 6). For DTs, RED achieves the lowest degree of bias in 13 out of 18 cases. Our post-processing approach is able to achieve the lowest degree of bias in 4 out 18 cases and the highest accuracy in 4 out of 6 cases. The pre-processing method LFR is never among the best performing methods for any of the four metrics (i.e., accuracy or fairness), while OP achieves the highest accuracy once for DTs on the COMPAS dataset. One reason that could explain the ability of RED to reduce bias further than pre-processing methods is that RED takes related fairness metrics into account. The pre-processing methods either re-balance the data or obfuscate sensitive information. These approaches are intuitive with regard to the overall goal of achieving fairness but do not coincide with the three measured fairness metrics.

Table 8 investigates the relation of the bias mitigation methods in a multi-objective setting, i.e., how often is our approach better than existing methods, how often is there a trade-off between accuracy and fairness, and how often is our method worse. From the results, we observe that our approach is comparable, if not better, than LFR and OP over all datasets and the two classification models (LR and DT). The same holds for RED applied to LR. However, REW tends to perform better than our approach for LR (pareto-optimal in 12 cases and better in 6).

Overall, we can observe that in the majority of the cases for LR (52 out of 66), our approach is pareto-optimal to existing pre- and in-processing approaches, indicating that there is a trade-off between fairness and accuracy. For practitioners, it would be important to consider more than one solutions to choose from, in particular those provided by our approach and REW, in order to select the best models with regards to specific datasets and metrics. For DT classifiers, we observe that our approach is strictly better than pre- and in-processing methods in 33 out of 66 cases, showing that there are performance difference among classification models. However, it is still beneficial to consider methods such as REW and RED, as there are cases in which they provide better results than our approach (i.e., they are strictly dominating).

In accordance with current findings (Pessach and Shmueli 2022), there is no single method that is the most suitable over all considered cases. Moreover, there is no clear preference on which stage bias mitigation methods should be applied. Rather, one has to take the dataset and fairness metric into account when selecting bias mitigation methods.

**Table 8:** RQ2-2: Comparison of our approach with pre- and in-processing methods in terms of domination criteria

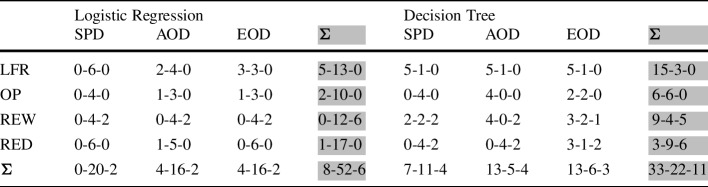	

For each of the four methods (three pre-processing, one in-processing) we provide results over the 6 datasets and three metrics as follows: our method dominates the existing method - both methods are pareto-optimal - our method is dominated. We determine domination with regard to accuracy and each of the three fairness metrics separately


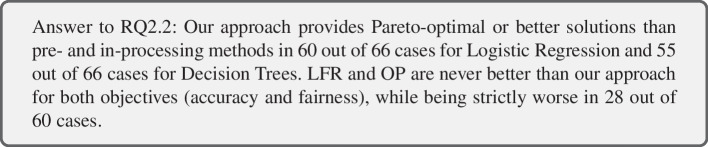


### RQ3. Impact on Fairness Metrics

In RQ3, we investigate the impact of optimizing for one fairness metric on the other two (e.g., if we optimize for accuracy and AOD, how do SPD and EOD change?). Therefore, we apply the three configurations of our post-processing approach on the four datasets and measure every kind of fairness metric at the end of the optimization procedure. In accordance with RQ1 and RQ2, we investigate the performance over 50 different train/validation/test splits.

**Fig. 3:**
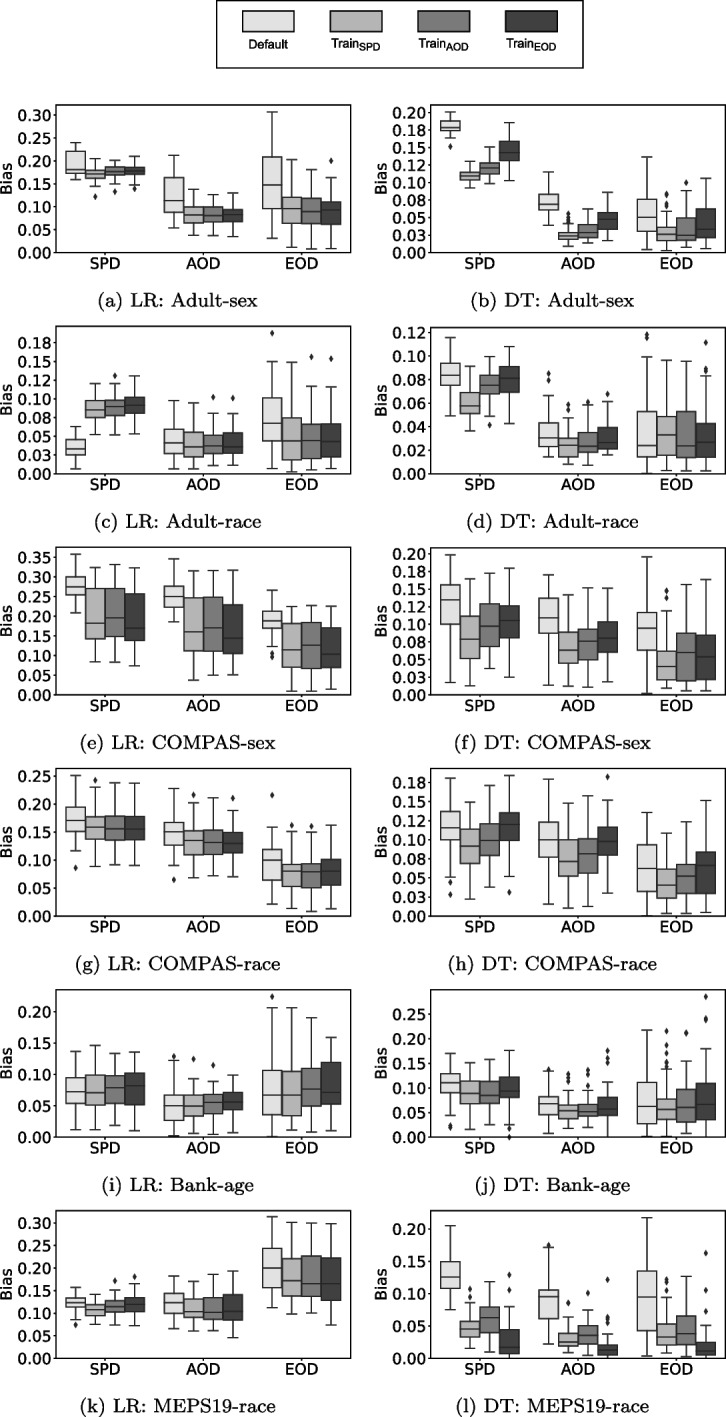
RQ3: Summary of bias values (the lower the better) achieved by the three different post-processing settings (SPD, AOD, EOD) and the default classification models. Boxplots are grouped based on the fairness metric they measure

Figure 3 shows the results of the optimization results. For each dataset, we use boxplots to show the default performance of the classification model, as well as the performance after optimization with each of the three configurations. Thereby, three colors represent optimization with one of the fairness metrics, and one color represents the fairness of the default classification model.

Given the results, we can see that the fairness achieved by an optimized, post-processed classification model behaves similarly, independent of the fairness metric used for optimization. For example, this can be seen on the Adult-sex dataset for LR and DT. Regardless of the fairness metric considered during optimization, the average AOD of all three configurations is better than the default classification model. Such a behaviour (all three optimization configurations achieve improvements on a fairness metric) happens in 28 out of 36 cases. There is one case (Adult-race for LR) in which none of the three search configurations achieve improvements on SPD (neither $LR_{SPD}$, $LR_{AOD}$ nor $LR_{EOD}$).

In the remaining 7 out of 36 cases, there are differences when using different optimization configurations. One example for this is the Bank-age datasets for LR. Only $LR_{SPD}$ achieves improvements over the default LR model in SPD, AOD and EOD. $LR_{AOD}$ and $LR_{EOD}$ are not able to improve any fairness metric (neither SPD, AOD or EOD).

To evaluate the overall level of bias mitigation achieved by optimization on a different fairness metric, we summarize the statistical significance differences we found over the four datasets in Table 9. In particular, we investigate whether significant improvements over the default classification models are achieved (*win*), whether no significant differences can be found (*tie*), or whether the default classification model has a statistically significant lower bias than the optimized model (*loss*). Combining the results for LR and DT, there are 45 wins, 24 ties and 3 losses. This indicates, that while our post-processing approach optimizes for one fairness metric, it can positively effect other metrics as well.

**Table 9:** RQ3: Win-tie-loss summary of the Wilcoxon tests when optimizing for one fairness metric and measuring the other two (e.g., use SPD during optimization and test on EOD) in comparison to the default classification model

	SPD		AOD		EOD		$\Sigma$
	AOD	EOD	SPD	EOD	SPD	AOD	
LR	4-2-0	4-1-1	3-3-0	4-2-0	3-3-0	4-2-0	22-13-1
DT	5-1-0	3-2-1	6-0-0	3-3-0	3-2-1	3-3-0	23-11-2

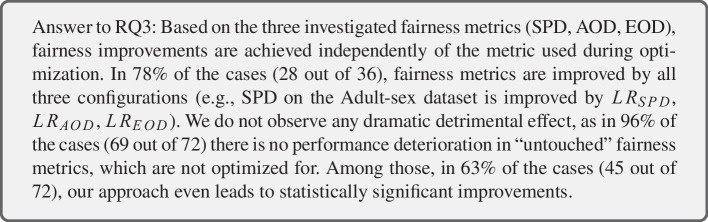



### Parameter Analysis for Logistic Regression

This section presents a closer investigation of parameter choices for our optimization procedure. An investigation of parameter choices is of particular importance for our experiments with Logistic Regression models, as the mutation operators are non-deterministic. In detail, we are interested in investigating the effect of the noise considered when modifying Logistic Regression models and the consideration of different terminal conditions (i.e., stopping the optimization process after a different number of steps) for three mutation types:

- **Reduction**: Multiply a single vector element by a random value within a range of $\{-noise, noise\}$.
- **Adjustment**: Multiply a single vector element by a random value within a range of $\{1-noise, 1+noise\}$.
- **Vector**: Multiply each vector element by a random value within a range of $\{1-noise, 1+noise\}$.

We investigate a total of three different levels of noise for mutation (0.05, 0.1, 0.2). While an increased number of steps should always be beneficial for improving a classification model (i.e., the chance of finding more fairness and accuracy improvements is higher), the question is whether the additional costs are justified. For this purpose, we consider three terminal conditions: 1000, 2500 and 5000 steps.

**Fig. 4:**
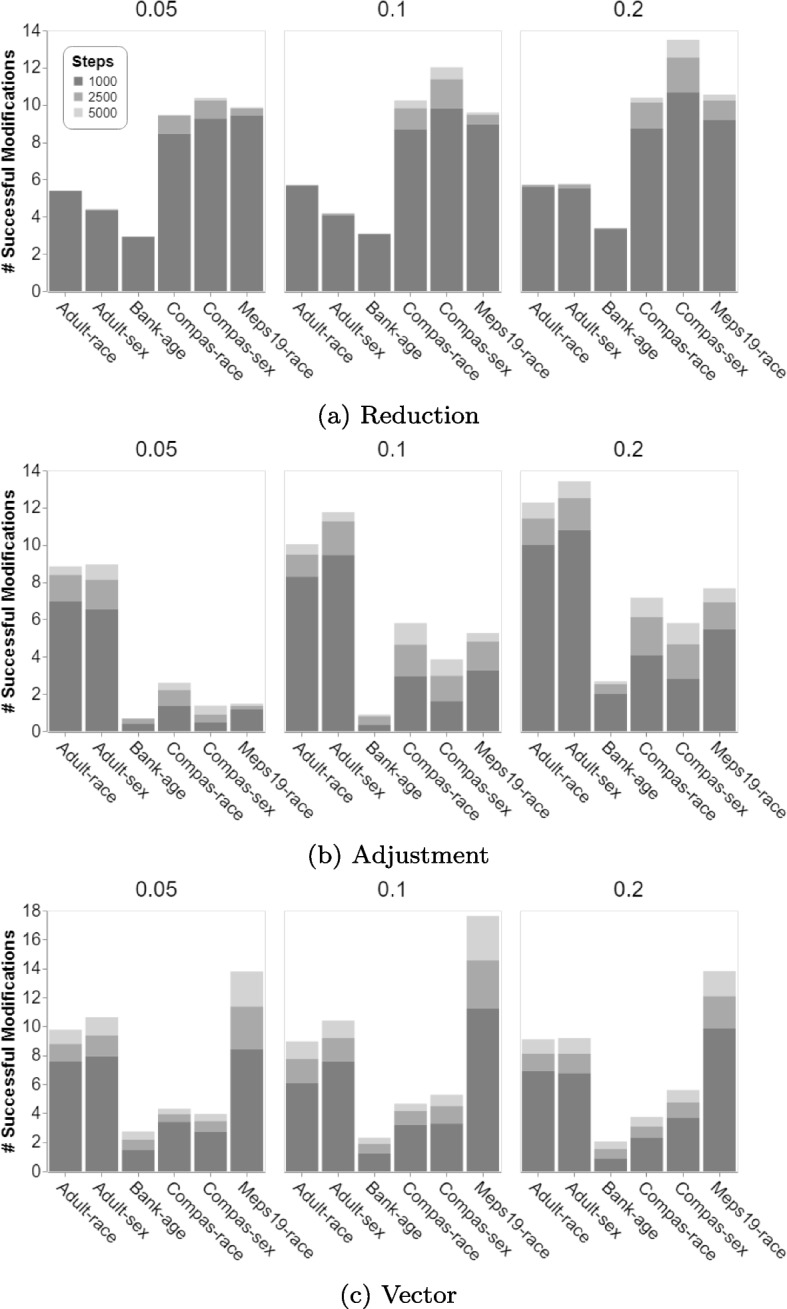
Average number of successful modifications of Logistic Regression model when applying our approach with three different noise degrees (0.05, 0.1, 0.2) after 1000, 2500 and 5000 steps. Values are averaged over 50 data-splits and three fairness metrics for optimization (SPD, AOD, EOD)

Figure 4 compares the number of successful modifications achieved by modifying Logistic Regression models with different degrees of noise, as well as the benefit of performing additional steps in the optimization procedure for the three mutation operators (*Reduction*, *Adjustment*, *Vector*). For the two mutation operators that modify a single element, *Reduction* and *Adjustment*, we can observe that the highest number of successful modifications is achieved by a mutation weight of 0.2. Among the 36 cases (two mutation operators $\times$ six datasets $\times$ three terminal conditions), there is only one case where a mutation weight of 0.1 achieves a higher number of successful mutations (i.e., 5.67 with a weight of 0.1 over 5.62 with a weight of 0.2, with *Reduction*). Using a mutation weight 0.2 for *Vector* modifications only achieves the highest number of successful modification for one of the six datasets (Compas-sex). Given that *Vector* modifications are more intrusive than the other mutation operators (i.e., modifying each vector element as opposed to modifying a single one), changes might be too big, or a stage where no further changes are applicable is reached quicker with high-noise modifications.

When applying *Reduction* modifications, an average 92.9% of all successful modification are performed in the first 1000 steps. Within an additional 1500 steps (i.e., terminal condition of 2500 steps), 5.6% of successful modification are performed. Only 1.6% of all successful modifications are performed in the last 2500 steps, from 2501 to 5000. While the percentages vary over datasets (e.g., after 1000 steps, 98% and 85% of modifications are performed for the Adult and COMPAS dataset respectively), it can be seen that the benefit of additional steps decreases over time, as the majority of modifications are performed within the first 1000 steps. *Vector* and *Adjustment* show similar results. The last 2500 steps (from 2501 to 5000) performed 10-15% of the modifications, while more than 60% of successful modifications are performed in the first 1000 steps. This confirms that the early steps of the optimization procedure are of higher importance than later iterations.

Given the low amount of additional modification achieved after 5000 steps, it is appears justified to not increase the limit for modifying Logistic Regression models further for our experiments (RQ1-RQ3), with the chances of potential improvements when using a mutation weight of 0.2. However, one could argue for decreasing the number of steps to 1000, which would decrease the runtime of our algorithm while retaining at least 60% of the successful modifications, depending on the mutation operator.

**Fig. 5:**
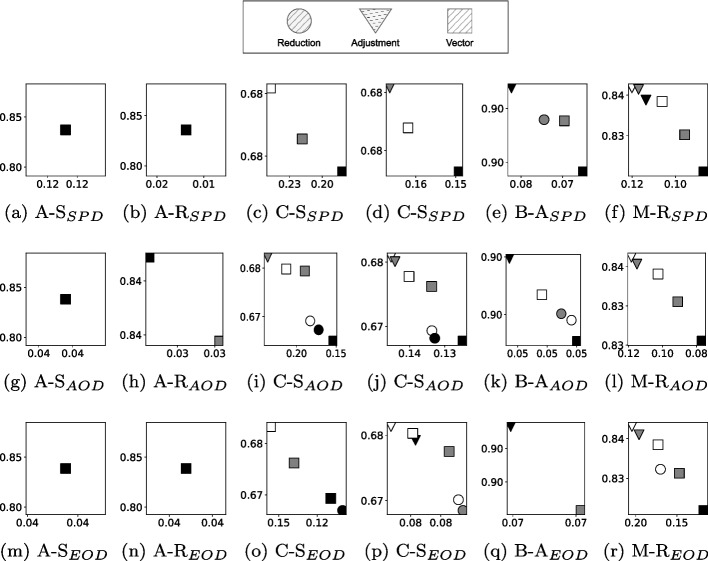
Pareto-fronts of the three different mutation operators (*Reduction*, *Adjustment*, *Vector*), and three levels of noise (0.2 - black, 0.1 - gray, 0.05 - white). Results are shown for four datasets: Adult (A), COMPAS (C), Bank (B), MEPS19 (M). Three protected attributes are considered: race (R), sex (S), age (A). The y-axis shows accuracy; the x-axis shows the respective fairness metric

Lastly, we compare the quality of changes between the three mutation operators. This allows us to not only compare the amount of modifications but also the effectiveness of different operators. For this purpose, we illustrate the pareto-fronts for each of the fairness metrics in combination with the achieved accuracy in Figure 5. Among the nine mutated LR models (three mutation operators with three different levels of noise, after 5000 steps), we only visualize non-dominated ones. The modification operator that is part of the most pareto-fronts is a *Vector* modification with a noise level of 0.2 (in 16 out of 18 pareto-fronts). *Reduction* and *Adjustment* are part of three to six pareto-fronts, depending on the level of noise used. This illustrates that the quality of improvements is influenced by the choice of mutation operators.

### Advanced Classification Models

Commonly, the effectiveness of bias mitigation methods is evaluated for a given classification model (e.g., which bias mitigation method should be applied to the model) rather than to compare performances across models (e.g., which model should the bias mitigation methods be applied to). Nonetheless, it can be interesting to compare the performance of more advanced binary classification models for potential future applications. For this purpose, we consider three advanced types of tree-based and regression-based classification models: Random Forest (RF), Gradient Boosting (GB), Neural Network (NN).

**Table 10:** Accuracy of Logistic Regression and Decision Tree approaches in comparison with advanced classification models. The highest accuracy for each dataset is highlighted in bold

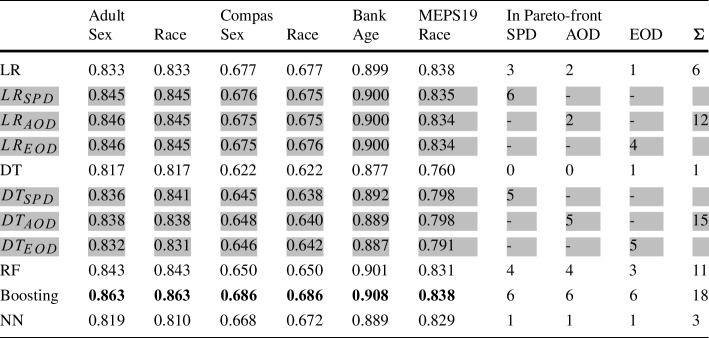	

Following existing fairness approaches (Chen et al. 2023b), our NN model consists of five hidden layers (64, 32, 16, 8, 4, neurons respectively) and is trained for 20 epochs. In accordance with our implementation of LR and DT models, RF and GB are implemented using the default configurations provided by scikit (Pedregosa et al. 2011).

Table 10 presents the accuracy achieved by each of the advanced classification models, Logistic Regression and Decision Trees, and our post-processing approach applied to both these models. To take fairness metrics in account, we count how often each classification model is part of any of the 18 fairness-accuracy pareto-fronts (six datasets and three fairness metrics), which illustrates trade-offs between fairness and accuracy.

Among all classification models, GB achieves the highest accuracy on all datasets, and outperforms RFs and NNs. NNs are outperformed by unmodified LR models for all datasets. RFs are outperformed by our optimized LR models in 5 out of 6 cases for accuracy, except for the Bank dataset. While DTs have the lowest accuracy, they also show the lowest degree of bias in 15 out of 18 cases. The only dataset for which DTs do not achieve the lowest degree of bias is the Bank dataset. For all three fairness metrics, NNs achieve the lowest degree of bias for the Bank dataset. This suggests, that it can be beneficial to carefully investigate and select suitable classification models for each use case.

Moreover, we observe that there is a trade-off between accuracy and fairness, as the classification model with the highest accuracy is never the one with lowest bias and vice versa. Nonetheless, it can be promising to use Boosting models as a starting point to apply bias mitigation to, as they exhibited the highest accuracy.

## Conclusions and Future Work

We proposed a novel search-based approach to mutate classification models in a post-processing stage, in order to simultaneously repair fairness and accuracy issues. This approach differentiates itself from existing bias mitigation methods, which conform to the fairness-accuracy trade-off (i.e., repair fairness issues come at a cost of a reduced accuracy). We performed a large scale empirical study to evaluate our approach with two popular binary classifiers (Logistic Regression and Decision Trees) on four widely used datasets and three fairness metrics, publicly available in the popular IBM AIF360 framework (Bellamy et al. 2018).

We found that our approach is able to simultaneously improve accuracy and fairness of both classification models in 61% of the cases. Our approach is particularly effective for Decision Trees, where we achieve statistically significant improvement on both accuracy and fairness in 81.1% of the cases. Moreover, we achieved improvements without detrimental effect on other fairness metrics that are not considered during optimization.

The comparison with three existing post-processing bias mitigation methods showed that none of these methods is able to achieve an accuracy as high as our method in any of the datasets. Furthermore, our approach is able to outperform existing post-processing methods in both accuracy and fairness in 12 out of 18 cases for Decision Trees.

These findings show not only the feasibility but also the effectiveness of our approach with respect to existing bias mitigation methods. Software engineers would benefit to have this tool at their disposal when developing fair software, as it allows them to find good trade-offs between competing objectives rather than proposing a solution which often sacrifices accuracy, as done in previous work. According to their needs, engineers can choose the solution that better conforms to their fairness and accuracy constraints.

The promising results reported herein can be further strengthened in future work. In particular, while we already investigated two inherently different classification models (Logistic Regression and Decision Trees) and various mutation operators, it could be of interest to further extend our approach to other binary classification models (e.g., Neural Network, Gradient Boosting) and mutation operators, as these could lead to further improvements in the results, as highlighted in Sections 5.4 and 5.5.

## Data Availability

We make our scripts and experimental results publicly available to allow for replication and extension of our work (Hort et al. 2023d).

## Footnotes

1 In this paper, we use term “bias repair” and “bias mitigation” alternatively to refer to the activities conducted to improve software fairness.

2 Decision-making scenarios that highly demand fairness often require high explainability, while low explainability is a big disadvantage of big complex models such as Deep Neural Networks.

3 https://meps.ahrq.gov/mepsweb/

4 We have performed a comparison of different data splits (i.e., it is beneficial to train with more data by combining train and validation) set but could not find systematic advantages. Further details can be found in our online appendix (Hort et al. 2023d).

5 There is no particular reason for choosing to run it 30 times, this number can be adjusted as one sees fit. Ideally the more runs the better, in order to cater for the inherent stochastic nature of the approach, yet limited computational resources or time may limit the number of repetitions performed. In practice, only one classification model can be used, therefore one can apply our approach multiple times and select a model from the Pareto-front, or use the entire search budget on building a single optimal classification model.

6 This is the set of solutions that are non-dominated to each other but are superior to the rest of solutions in the search space. In other words each solution of the Pareto-set includes at least one objective inferior to another solution in that Pareto-set, although both solutions are superior to others in the rest of the search space with respect to all objectives.

7 Here we use $K=12$, for the two hypothesis and the six datasets. In Tables 2 and 3, we report the original p-value (i.e., with no correction) so that a reader could assess the results using a different correction, if interested.

8 https://aif360.mybluemix.net/
